# The influence of mode of delivery on neonatal and maternal short and long-term outcomes

**DOI:** 10.11606/S1518-8787.2018052000742

**Published:** 2018-11-27

**Authors:** Daniela Siqueira Prado, Rosemar Barbosa Mendes, Rosana Queiroz Gurgel, Ikaro Daniel de Carvalho Barreto, Rosana Cipolotti, Ricardo Queiroz Gurgel

**Affiliations:** 1 Universidade Federal de Sergipe. Departamento de Medicina. Aracaju, SE, Brasil; 2 Universidade Federal de Sergipe. Departamento de Enfermagem. Aracaju, SE, Brasil; 3 Universidade Tiradentes. Departamento de Enfermagem. Aracaju, SE, Brasil; 4 Universidade Federal Rural de Pernambuco. Pós-graduando do Programa de Biometria e Estatística Aplicada. Recife, PE, Brasil

**Keywords:** Cesarean Section, Delivery, Obstetric, Breast Feeding, Depression, Postpartum, Urinary Incontinence, Sexual Dysfunction, Physiological, Cohort Studies

## Abstract

**OBJECTIVE:**

To evaluate the impact of mode of delivery on breastfeeding incentive practices and on neonatal and maternal short and long-term complications.

**METHODS:**

A cohort study was conducted between June 2015 and April 2016 with 768 puerperal women from 11 maternities in Sergipe, interviewed in the first 24 hours, 45–60 days and 6–8 months after delivery. Associations between breastfeeding incentive practices, neonatal and maternal, both short-term and late complications, and the exposure variables were evaluated by the relative risk (95%CI) and the Fisher exact test.

**RESULTS:**

The C-section newborns had less skin-to-skin contact immediately after delivery (intrapartum C-section: 0.18, 95%CI 0.1–0.31 and elective C-section: 0.36, 95%CI 0.27–0.47) and less breastfeeding within one hour of birth (intrapartum C-section: 0.43, 95%CI 0.29–0.63 and elective C-section: 0.44, 95%CI 0.33–0.59). Newborns from elective C-section were less frequently breastfed in the delivery room 0.42 (95%CI 0.2–0.88) and roomed-in less 0.85 (95%CI 0.77–0.95). Women submitted to intrapartum C-section had greater risk of early complications 1.3 (95%CI 1.04–1.64, p = 0.037) and sexual dysfunction 1.68 (95%CI 1.14–2.48, p = 0.027). The frequency of neonatal complications, urinary incontinence and depression according to the mode of delivery was similar.

**CONCLUSIONS:**

The C-section was negatively associated with breastfeeding incentive practices; in addition, C-section after labor increased the risk of early maternal complications and sexual dysfunction.

## INTRODUCTION

The cesarean section (C-section) is the most frequently performed surgical procedure in reproductive-aged women^1^. The C-section rates are increasing worldwide^2^, and Brazil is among the leaders, with 51.9% of Brazilian births using a C-section; this figure reaches 88% in the private health sector^3^.

With rising C-section rates, studies have shown the dangers of this procedure to both the mother and the newborn. A prospective multicenter study with eight Latin American countries showed a higher rate of morbidity in women that opted for a C-section when compared with vaginal delivery on the following events: hysterectomy, length of hospital permanence, and use of antibiotic therapy. For the newborn, the neonatal mortality rate in elective C-sections with cephalic presentations decreased slightly and in those with pelvic presentation reduced significantly. However, C-sections were associated with a higher risk of hospitalization for over seven days in the neonatal ICU and neonatal death after discharge^4^. Later in infancy, the C-section is associated with a higher rate of asthma, allergic rhinitis, atopy, type 1 diabetes mellitus, and celiac disease^1^.

In contrast to the described findings, a Chinese cohort study found no difference between patients that underwent a vaginal delivery or a C-section per request regarding maternal morbidity, and C-section per request was associated with fewer newborn trauma in the moment of delivery, as well as fewer neonatal infection, hypoxic-ischemic encephalopathy, and meconium aspiration. In contrast, vaginal delivery has been associated with fewer neonatal respiratory disorders. The results for operatory vaginal delivery and intrapartum C-sections were worse than those for vaginal delivery and on-demand C-section^5^.

The studies are controversial regarding sexual dysfunction, depression, and postpartum urinary incontinence. Some studies show a lower sexual satisfaction and higher frequencies of urinary incontinence and depression in women that had vaginal delivery when compared with C-section^6^, while other studies show no difference^7^ ^–^ ^9^.

Findings differ depending on the geographical location. A recent statement from the World Health Organization (WHO) reports that the effects of the C-section on outcomes beyond mortality (e.g., maternal and perinatal morbidity, pediatric outcomes, social or psychological well-being) are unclear. Further studies are needed to understand the short- and long-term effects of the C-section procedure^10^.

The epidemic of unnecessary caesareans is a global concern. This study aimed to evaluate the repercussions of the type of delivery on neonatal and maternal outcomes of puerperal women who attended the 11 maternities of a Northeastern Brazilian state.

## METHODS

### Design and Settings

We conducted a cohort study between June 2015 and April 2016 at all the maternities with more than 500 births/year in the Brazilian state of Sergipe. The study method of “Birth in Brazil” was reproduced, and researchers from the national study trained the local team^3^.

The sample calculation estimated the prevalence of C-section in Sergipe, for institutions with over 500 births/year with a 95% confidence interval, using the C-section rate of 38% (DATASUS, 2011)^11^, amounting to 358 women to be interviewed. Considering the possibility of losses in the follow-up, 768 puerperae were evaluated. A proportional allocation, according to each institution's dimension, was adopted to distribute the sample size.

Each hospital had patients included for a minimum of seven days to complete a full working week. If the number of individuals were to be reached before this period, a random limiting number of daily interviews was set to reach the seven days period.

### Study Population

Patients whose baby's weight was over 500 g or gestational age was higher than 22 weeks were included in the study. The exclusion criteria were not speaking or understanding Portuguese as well as the impossibility of phone contact after delivery. All eligible puerperal women agreed to participate in the study.

### Data Collection

Face-to-face interviews were carried out with the 768 puerperal women between 6h and 24h after delivery, and we extracted data from the woman's and the newborn's records after discharge (or death). In cases of prolonged hospital permanence, the puerperal women's data were extracted after 42 days of hospitalization; and the newborns' data, after 28 days.

Follow-up telephone interviews were conducted at 45–60 days to investigate early neonatal and maternal complications and at six to eight months postpartum to investigate sexual dysfunction, depression, and urinary incontinence through the application of three questionnaires validated in Brazil: The Female Sexual Function Index (FSFI)^12^; the International Consultation on Incontinence Questionnaire - Short Form (ICIQ-SF)^13^, and the Edinburgh Postnatal Depression Scale (EPDS)^14^.

During the 45–60-day period, there was a loss of follow-up of 210 (27.3%) women, and of 450 (58%) during the 6–8-month period.

### Variables and Definitions

The exposure variables studied were: type of delivery- vaginal, cesarean section after labor (intrapartum), and cesarean section without labor (elective); source of funding- public (by the Brazilian Unified Health System) or private (by the patient or health care insurance); hospital with baby-friendly certification or not^15^; and habitual obstetric risk or high risk (gestational diabetes, hypertension, obesity, acquired immunodeficiency syndrome, less than 37 weeks of gestational age or more than 41 weeks at birth, multiple pregnancy, non-cephalic presentations, birth weight lower than 2,500 g or above 4,499 g, and below the 5th percentile or above the 95th percentile of weight per gestational age)^16^.

The outcomes evaluated were: breastfeeding incentive practices (early skin-to-skin contact after delivery, breastfeeding in the delivery room, breastfeeding within one hour of birth and mother-infant rooming-in during hospitalization)^15^; adverse neonatal outcome, including neonatal death (within the first 28 days of life), birth weight below 1,500 g, Apgar score at five minutes below seven, need for mechanical ventilation, and less than 32 weeks of gestational age. Minor neonatal complications included transient tachypnea, meconium aspiration syndrome and hyaline membrane disease. Early maternal complications included fever, hemorrhage, need for blood transfusion, pain in the perineum or cesarean scar, and need for puerperal hysterectomy. Late maternal complications included sexual dysfunction (IFSF score lower than 26.5), urinary incontinence (qualified and quantified by the ICIQ-SF), and depression (EPDS score higher than 10).

### Statistical Analysis

For statistical analysis, simple frequencies and percentages described associations between breastfeeding incentive practices, major and minor neonatal complications, and early and long-term maternal complications with exposure variables, in addition to relative risk with 95% confidence intervals and Fisher's exact test for associations between categorical variables. The R Core Team 2017 software was used. A p value of < 0.05 was considered significant.

### Ethical Approval

The Ethics in Research Committee of the Universidade Federal de Sergipe approved the protocol (CAAE 22488213.4.0000.5546). All puerperal women signed an informed consent form with the guarantee of refusal at any moment.

## RESULTS

The average age of the participants was 25 years. For education, 46.4% had seven or less years of study. The majority had no paid work (55.5%) and lived with their partner (84.3%). Regarding parity, 54,9% were multiparas. The type of delivery was vaginal in 59.4% of cases and C-section in 40.5%. Regarding the type of financing, the C-section rate was 35% in public services and 75% in private services (Table 1).

**Table 1:** Sociodemographic characteristics and obstetric aspects of the study population. Sergipe, Brazil, June 2015 to April 2016.

Characteristic		n (%)
Mean maternal age (SD)		25.35 (6.52)
	10-19	164 (21.4)
	20-34	532 (69.3)
	≥ 35	72 (9.4)
Race/Ethnicity		
	White	109 (14.2)
	Nonwhite	659 (85.8)
Years of study		
	≤ 7	356 (46.4)
	8-10	310 (40.4)
	≥ 11	101 (13.2)
Paid work		
	No	426 (55.5)
	Yes	342 (44.5)
Marital status		
	With partner	647 (84.3)
	Without partner	120 (15.6)
Parity,		
	0	37 (4.8)
	1-2	309 (40.2)
	≥ 3	422 (54.9)
Mode of delivery		
	Vaginal	456 (59.4)
	Intrapartum cesarean section	110 (14.3)
	Elective cesarean section	202 (26.3)

Early skin-to-skin contact after delivery occurred in 41% of cases, breastfeeding in the delivery room was offered to 7.8% of newborns, 33.1% were breastfed in the first hour after delivery, and 75.1% roomed-in with their mothers during the entire hospitalization. Adverse neonatal outcome was demonstrated in 6% of the cases – 1.5% had neonatal deaths, 1.2% had weight < 1,500 g, 1.3% had Apgar score at the 5th minute < 7, 3.2% needed mechanical ventilation, and 2.1% had gestational age < 32 weeks. Minor neonatal complications reported corresponded to 2.6% – transient tachypnea in 2%, hyaline membrane disease in 0.4%, and meconium aspiration syndrome in 0.4%. Early maternal complications occurred in 46.8%, and pain was the most frequent, (46.1% in the perineum and 46.7% in the cesarean operative wound) followed by high fever (26.3%) and severe bleeding (15.5%). Transfusion or puerperal hysterectomy was not needed in the studied population. The frequency of sexual dysfunction, urinary incontinence, and postpartum depression was 36.4%, 11.8%, and 19.1%, respectively.

The C-section newborns had less skin-to-skin contact with their mothers immediately after delivery (intrapartum C-section: RR = 0.18, 95%CI 0.1–0.31 and elective C-section: RR = 0.36, 95%CI 0.27–0.47) and had less breastfeeding within one hour of birth (intrapartum C-section: RR = 0.43, 95%CI 0.29–0.63 and elective C-section: RR = 0.44, 95%CI 0.33–0.59) when compared with vaginal delivery newborns (Table 2). Elective C-section newborns had less breastfeeding in the delivery room RR = 0.42 (95%CI 0.2–0.88), and they also roomed-in less RR = 0.85 (95%CI 0.77–0.95) when compared with vaginal delivery and intrapartum C-section (Table 2). In hospitals certified as baby-friendly, skin-to-skin contact (RR = 1.51, 95%CI 1.27–1.8, p < 0.001) and rooming-in (RR = 1.32, 95%CI 1.21–1.43, p < 0.001) were more frequent (Table 2).

**Table 2:** Breastfeeding incentive practices according to type of delivery. Sergipe, Brazil, June 2015 to April 2016.

Variable	Skin-to-skin contact after delivery			Breastfeed in the delivery room			Breastfeed within one hour of birth			Rooming-in		
	n (%)	RR (95%CI)	p	n (%)	RR (95%CI)	p	n (%)	RR (95%CI)	p	n (%)	RR (95%CI)	p
Global												
Intrapartum C-section	11 (10.2)	0.18 (0.10-0.31)	< 0.001	7 (6.8)	0.64 (0.30-1.38)	0.275	21 (23.1)	0.43 (0-29-0.63)	< 0.001	81 (73.6)	0.93 (0.83-1.05)	0.250
Elective C-section	42 (20,8)	0.36 (0.27-0.47)	< 0.001	8 (4.5)	0.42 (0.20-0.88)	0.017	39 (23.9)	0.44 (0.33-0.59)	< 0.001	136 (67.3)	0.85 (0.77-0.95)	0.002
Normal	262 (58.0)	1		45 (10.6)	1		194 (53.7)	1		355 (78.9)	1	
Public Low-Risk												
Intrapartum C-section	5 (10.6)	0.18 (0.08-0.42)	< 0.001	3 (7.1)	0.68 (0.22-2.13)	0.783	11 (30.6)	0.56 (0.34-0.93)	0.008	36 (76.6)	0.93 (0.79-1.10)	0.418
Elective C-section	9 (12.2)	0.21 (0.11-0.39)	< 0.001	5 (7.1)	0.68 (0.27-1.68)	0.507	19 (30.2)	0.55 (0.37-0.82)	0.001	58 (78.4)	0.95 (0.84-1.08)	0.504
Normal	179 (58.5)	1		31 (10.5)	1		137 (54.6)	1		251 (82.3)	1	
Private Low-Risk												
Intrapartum C-section	1 (7.7)	0.09 (0.01-0.61)	< 0.001	0 (0)	*	0.498	0 (0)	*	0.225	10 (76.9)	1.38 (0.83-2.30)	0.275
Elective C-section	21 (45.7)	0.52 (0.35-0.77)	0.005	2 (4.8)	0.40 (0.06-2.64)	0.571	2 (5.0)	0.23 (0.04-1.25)	0.103	30 (65.2)	1.17 (0.74-1.87)	0.569
Normal	15 (83.3)	1		2 (11.8)	1		3 (21.4)	1		10 (55.6)	1	
Public High-Risk												
Intrapartum C-section	4 (10.5)	0.20 (0.08-0.50)	< 0.001	3 (7.7)	0.70 (0.21-2.37)	0.760	9 (26.5)	0.46 (0.26-0.83)	0.003	27 (67.5)	0.92 (0.72-1.17)	0.544
Elective C-section	5 (7.8)	0.15 (0.06-0.34)	< 0.001	1 (1.9)	0.18 (0.02-1.32)	0.063	18 (38.3)	0.67 (0.45-0.99)	0.049	39 (60.9)	0.83 (0.66-1.04)	0.096
Normal	67 (53.6)	1		12 (10.9)	1		54 (57.4)	1		91 (73.4)	1	
Private High-Risk												
Intrapartum C-section	1 (10)	0.30 (0.03-3.49)	0.423	1 (10)	*	1.000	1 (11.1)	* 1.000		8 (80)	0.80 (0.57-1.09)	1.000
Elective C-section	7 (38.9)	1.17 (0.21-0.77)	1.000	0 (0)	*		0 (0)	*		9 (50)	0.50 (0.31-0.79)	0.229
Normal	1 (33.3)	1		0 (0)	*		0 (0)	*		3 (100)	1	
Baby-friendly hospitals												
Yes	186 (50.0)	1.51 (1.27-1.80)	< 0.001	31 (8.8)	1.07 (0.66-1.74)	0.789	126 (40.8)	0.97 (0.81-1.18)	0.806	318 (85.7)	1.32 (1.21-1.43)	< 0.001
No	129 (33.1)	1		29 (8.2)	1		128 (41.8)	1		254 (65.0)	1	

RR: relative risk; p-value: Fisher's exact test

* Relative risk was not evaluated by numerical issues

The frequency of adverse neonatal outcomes depending on the delivery type was similar (Table 3).

**Table 3:** Adverse neonatal outcome (major complications), minor neonatal complications and early maternal complications according to type of delivery. Sergipe, Brazil, June 2015 to April 2016.

Variable	Adverse neonatal outcome			Minor neonatal complications			Early maternal complications		
	n (%)	RR (95%CI)	p	n (%)	RR (95%CI)	p	n (%)	RR (95%CI)	p
Global									
Intrapartum C-section	7 (6.4)	1.04 (0.46-2.31)	1.000	2 (1.8)	0.83 (0.18-3.73)	1.000	47 (59.5)	1.30 (1.04-1.64)	0.037
Elective C-section	11 (5.4)	0.89 (0.45-1.75)	0.858	8 (4.0)	1.81 (0.72-4.51)	0.204	63 (42)	0.92 (0.73-1.16)	0.526
Normal	28 (6.1)	1		10 (2.2)	1		104 (45.6)	1	
Public low-risk									
Intrapartum C-section	1 (2.1)	0.65 (0.09-5.00)	1.000	2 (4.3)	6.55 (0.95-45.4)	0.087	22 (61.1)	1.17 (0.87-1.59)	0.356
Elective C-section	1 (1.4)	0.42 (0.05-3.20)	0.699	1 (1.4)	2.08 (0.19-22.6)	0.477	19 (38)	0.73 (0.50-1.07)	0.102
Normal	10 (3.2)	1		2 (0.6)	1		77 (52)	1	
Private low-risk									
Intrapartum C-section	1 (7.7)	1.38 (0.09-20.2)	1.000	0 (0)	a	b	2 (28.6)	1.24 (0.27-5.75)	1.000
Elective C-section	1 (2.2)	0.39 (0.03-5.93)	0.487	1 (2.2)	a	1.000	14 (35.9)	1.55 (0.53-4.57)	0.506
Normal	1 (5.6)	1		0 (0)	1		3 (23.1)	1	
Public high-risk									
Intrapartum C-section	3 (7.5)	0.56 (0.17-1.84)	0.411	0 (0)	a	0.200	18 (62.1)	1.65 (1.08-2.53)	0.042
Elective C-section	8 (12.5)	0.93 (0.43-2.05)	1.000	5 (7.8)	1.24 (0.42-3.64)	0.764	21 (43.8)	1.17 (0.74-1.83)	0.562
Normal	17 (13.4)	1		8 (6.3)	1		24 (37.5)	1	
Private high-risk									
Intrapartum C-section	2 (20)	a	1.000	0 (0)	a	b	5 (71.4)	a	0.167
Elective C-section	1 (5.6)	a	1.000	1 (5.6)	a	1.000	9 (69.2)	a	0.063
Normal	0 (0)	1		0 (0)	1		0 (0)	1	

RR: relative risk; p-value: Fisher's exact test

a Relative risk was not evaluated by numerical issues.^a^

b p was not evaluated by numerical issues.

Women undergoing intrapartum C-section had a higher risk of early complications (RR = 1.3, 95%CI 1.04–1.64, p = 0.037), more evident in the subgroup of public service high-risk puerperal women (RR = 1.65, 95%CI 1.08–2.53, p = 0.042) (Table 3).

Sexual dysfunction six to eight months after intrapartum C-section was more frequent than after vaginal delivery (RR = 1.68, 95%CI 1,14–2.48, p = 0.027) (Table 4), in the high-risk public service subgroup (RR = 2.19, 95%CI 1.27–3.72, p = 0.029) (Table 4). The frequency of urinary incontinence and puerperal depression according to the type of delivery was similar (Table 4).

**Table 4:** Long-term maternal complications (sexual dysfunction, urinary incontinence, and depression) by type of delivery. Sergipe, Brazil, June 2015 to April 2016.

Variable	Sexual dysfunction (IFSF < 26.5)			Urinary incontinence			Postdelivery depression (EDPS . 10)		
	n (%)	RR (95%CI)	p	n (%)	RR (95%CI)	p	n (%)	RR (95%CI)	p
Global									
Intrapartum C-section	18 (54.5)	1.68 (1.14.2.48)	0.027	8 (18.6)	1.49 (0.71.3.11)	0.324	7 (17.9)	0.84 (0.41.1.75)	0.827
Elective C-section	34 (40)	1.23 (0.87.1.74)	0.259	7 (7.4)	0.59 (0.26.1.33)	0.222	14 (15.6)	0.73 (0.42.1.28)	0.322
Normal	49 (32.5)	1		22 (12.5)	1		36 (21.3)	1	
Public low-risk									
Intrapartum C-section	5 (31.3)	0.99 (0.45.2.16)	1.000	3 (14.3)	1.17 (0.37.4.61)	0.727	3 (15.)	0.81 (0.26.2.45)	1.000
Elective C-section	14 (45.2)	1.43 (0.88.2.32)	0.197	2 (5.4)	0.44 (0.11.1.86)	0.360	6 (16.7)	0.90 (0.39.2.05)	1.000
Normal	31 (31.6)	1		14 (12.2)	1		21 (18.6)	1	
Private low-risk									
Intrapartum C-section	5 (71.4)	2.62 (0.90.7.66)	0.145	0 (0)	a	1.000	0 (0)	a	0.515
Elective C-section	8 (42.1)	1.54 (0.51.4.64)	0.466	1 (5.0)	0.50 (0.03.7.19)	1.000	1 (5.6)	0.31 (0.03.2.99)	0.539
Normal	3 (27.3)	1		1 (10)	1		2 (18.2)	1	
Public high-risk									
Intrapartum C-section	7 (77.8)	2.19 (1.27.3.72)	0.029	4 (33.3)	2.38 (0.83.6.83)	0.200	3 (30)	1.01 (0.35.2.90)	1.000
Elective C-section	11 (40.7)	1.14 (0.62.2.10)	0.800	3 (10.0)	0.71 (0.20.2.55)	0.736	5 (16.7)	0.56 (0.22.1.42)	0.273
Normal	15 (35.7)	1		7 (14.0)	1		13 (29.5)	1	
Private high-risk									
Intrapartum C-section	1 (100)	a		1 (33.3)	a	1.000	1 (33.3)	a	1.000
Elective C-section	1 (12.5)	a		1 (12.5)	a	1.000	2 (33.3)	a	1.000
Normal	0 (0)	a		0 (0)	1		0 (0)	a	

RR: relative risk; p-value: Fisher's exact test; IFSF: por extenso; EDPS: por extenso

a Relative risk was not evaluated by numerical issues

## DISCUSSION

In this study, neonatal and maternal outcomes were similar after elective cesarean section and vaginal delivery.

The definition of the safest and the best delivery method cannot be completely established yet. No randomized studies compared the results of cephalic fetuses of vaginal delivery *versus* cesarean section without medical indication^17^.

A systematic review of 54 papers found that a large portion of the studies had low methodological quality that prevented robust conclusions. Among the more consistent results, C-section is linked with an increased risk of placenta previa in future pregnancies, greater maternal hospital permanence, neonatal respiratory morbidity (which decreases with increasing gestational age at which the surgery is performed), and lower risk of hemorrhage and blood transfusion^18^. A meta-analysis of maternal complications associated with cesarean section without medical indication showed women with cesarean section have a higher chance of maternal death (OR = 3.10, 95%CI 1.92–5.00) and postpartum infection (OR = 2.83, 95%CI 1.58–5.06), but they have a lower chance of hemorrhage (OR = 0.52, 95%CI 0.48–0.57). Most of the studies included had retrospective secondary data from large health systems databases or medical record review. No prospective study was included^19^.

This study was conducted in the smallest Brazilian state (i.e., Sergipe), located in a socioeconomically poor region in Northeastern Brazil and showed a low frequency of practices to stimulate breastfeeding.

We verified less skin-to-skin contact in the delivery room and breastfeeding within one hour of birth after cesarean section. Patients undergoing an elective cesarean section breastfed less in the delivery room; also, their newborns roomed-in less. A study conducted in the United States showed association between less frequent breastfeeding practices and more frequent discontinuation of breastfeeding before six weeks after delivery^20^. A prospective Australian study also found that cesarean sections were associated with less frequent early skin-to-skin contact and breastfeeding within one hour after birth when compared with the vaginal route^21^.

There was no association between the delivery type and severe adverse neonatal outcome or minor neonatal complications. The percentage of minor complications was higher for infants born by elective C-section, including transient tachypnea, but without statistical significance. A Danish cohort study reported a higher risk of respiratory morbidity in neonates after elective C-section at 37 weeks (OR = 3.9, 95%CI 2.4–6.5), which was attenuated at 39 weeks (OR = 1.9, 95%CI 1.2–3.0) when compared with vaginal delivery^22^. However, a control-case study performed in the Southeastern Brazil with 27,252 newborns found no association between C-section delivery and Apgar scores < 7 at the 5th postpartum minute^23^.

In this study, the most frequent maternal complication was pain, which had a similar frequency after vaginal delivery or cesarean section. Although heavy puerperal bleeding was reported by 15.5% of the puerperal women, transfusion or hysterectomy was not needed in any case. Patients who had a C-section after labor showed a higher risk of short-term complications and sexual dysfunction than those who had vaginal delivery or elective C-section. In a study with nine Asian countries, patients undergoing intrapartum C-section had higher morbidity/mortality than those with antepartum-C-section, and all types of C-section had a higher risk of maternal morbidity/mortality when compared with vaginal delivery, except for perineal lacerations^24^.

One interesting cultural aspect of Brazil and many Latin American countries is the indication of C-section to avoid pelvic floor damage. In this study, the frequency of urinary incontinence according to the type of delivery was similar, although it was lower after elective C-section; this result is similar to that observed in a study conducted in the Southeast Brazil^8^. The frequency of sexual dysfunction was similar after vaginal delivery and elective C-section and somewhat higher after cesarean section preceded by labor. Depression was also not associated with the delivery type in this study; this result corroborates a prospective five-time evaluation study that demonstrated a higher frequency of post-C-section depression compared with vaginal delivery three months postpartum but no more at six months, and the sexual function score by type of delivery was similar^25^.

A limitation of this study was the loss of 27.3% of the cases in the follow-up evaluation at 45 to 60 days after delivery and of 58% at six to eight months postpartum; such losses were analyzed and were homogeneous according to delivery type. Another limitation was the lack of predelivery data on sexual dysfunction, urinary incontinence, and depression.

The prospective design, the large number of participants, and the use of validated questionnaires add credibility to this study. We could prospectively evaluate 768 postpartum women, which is superior to the number of most studies on the topic. The inclusion of participants from all maternities in Sergipe in a statistically adequate number allows generalize the results at a state level. The ideal study design to evaluate complications by delivery type would be a randomized clinical trial, but conducting such a study has ethical implications.

In conclusion, C-section was negatively associated with breastfeeding incentive practices. Adverse neonatal outcome and minor neonatal complications regarding delivery type were not associated. Intrapartum C-section was associated with a higher risk of short-term maternal complications and sexual dysfunction. There was no association between type of delivery and urinary incontinence and depression.
